# Supplementary figures and images for: Compatibility of endoscopic examination using i-scan technology with histopathology results in laryngeal carcinoma: prospective observational study
Source: PeerJ. 2025 Aug 7;13:e19552. doi: 10.7717/peerj.19552 (PMC12335829; doi:10.7717/peerj.19552)

| No | Name | WLE                                                                                 | L-SCAN 3                                                                            |
|----|------|-------------------------------------------------------------------------------------|-------------------------------------------------------------------------------------|
| 1  | AL   | 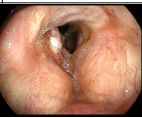   | 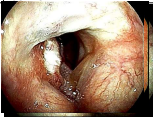   |
| 2  | YR   | 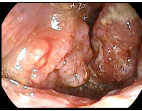   | 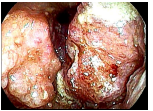   |
| 3  | SI   | 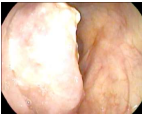   | 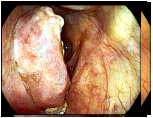   |
| 4  | AC   | 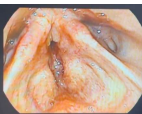   | 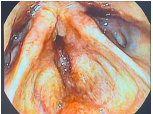   |
| 5  | AD   | 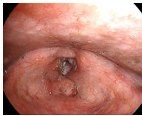   | 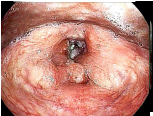   |
| 6  | OC   | 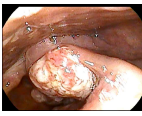   | 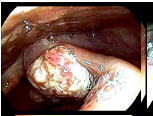   |
| 7  | RA   | 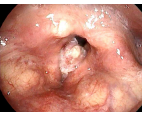  | 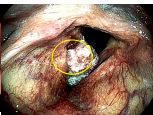  |
| 8  | TS   | 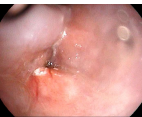 | 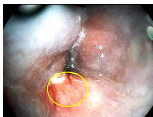 |
| 9  | EN   | 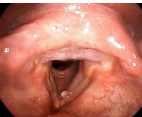 | 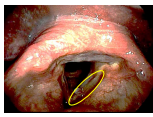 |
| 10 | ES   | 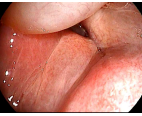 | 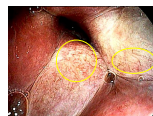 |
| 11 | MD   | 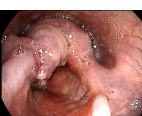 | 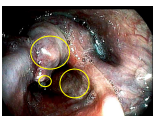 |
| 12 | IR   | 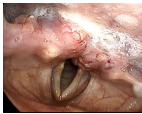 | 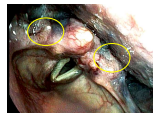 |

13 AF

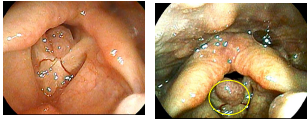

14 AG

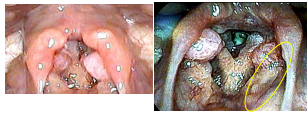

15 SH

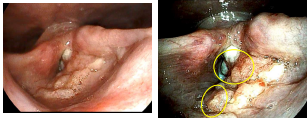

16 MS

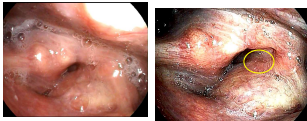

17 DS

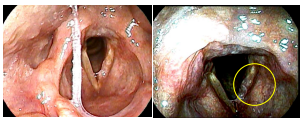

18 MR

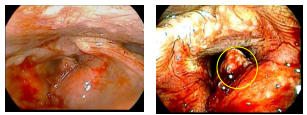

19 SM

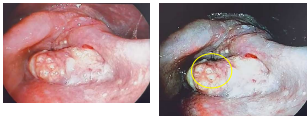

20 SS

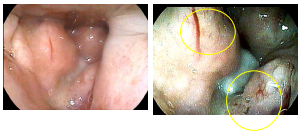

21 TA

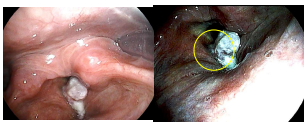

22 HH

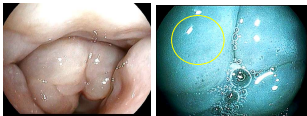

23 IP

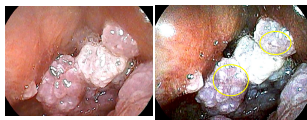

24 CR

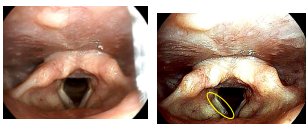

25 AN

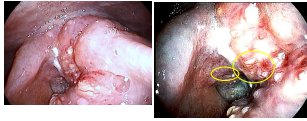

26 HE

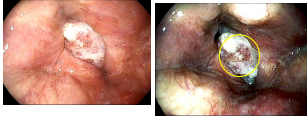

27 IH

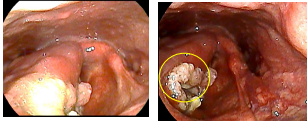

28 TK

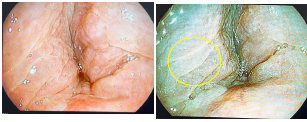

29 AS

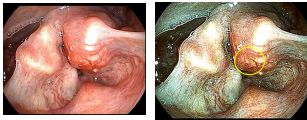

Supplement: Supplemental Information 2 — These images document the mucosal and vascular patterns of laryngeal lesions in all enrolled patients, as classified according to the Ni endoscopic system. The file serves as visual reference material supporting the diagnostic assessments and findings presented in the main manuscript. [file peerj-13-19552-s002.pdf]
